# microRNA-seq of cartilage reveals an overabundance of miR-140-3p which contains functional isomiRs

**DOI:** 10.1261/rna.075176.120

**Published:** 2020-11

**Authors:** Steven Woods, Sarah Charlton, Kat Cheung, Yao Hao, Jamie Soul, Louise N. Reynard, Natalie Crowe, Tracey E. Swingler, Andrew J. Skelton, Katarzyna A. Piróg, Colin G. Miles, Dimitra Tsompani, Robert M. Jackson, Tamas Dalmay, Ian M. Clark, Matt J. Barter, David A. Young

**Affiliations:** 1 Division of Cell Matrix Biology and Regenerative Medicine, Faculty of Biology Medicine and Health, University of Manchester, Manchester M13 9PT, United Kingdom; 2 Skeletal Research Group, Biosciences Institute, Newcastle University, Newcastle upon Tyne NE1 3BZ, United Kingdom; 3 Orthopedics Department, First Hospital of Shanxi Medical University, Yingze District, Taiyuan, 030000, China; 4 School of Biological Sciences, University of East Anglia, Norwich NR4 7TJ, United Kingdom

**Keywords:** cartilage, isomiR, microRNA

## Abstract

miR-140 is selectively expressed in cartilage. Deletion of the entire *Mir140* locus in mice results in growth retardation and early-onset osteoarthritis-like pathology; however, the relative contribution of miR-140-5p or miR-140-3p to the phenotype remains to be determined. An unbiased small RNA sequencing approach identified miR-140-3p as significantly more abundant (>10-fold) than miR-140-5p in human cartilage. Analysis of these data identified multiple miR-140-3p isomiRs differing from the miRBase annotation at both the 5′ and 3′ end, with >99% having one of two seed sequences (5′ bases 2–8). Canonical (miR-140-3p.2) and shifted (miR-140-3p.1) seed isomiRs were overexpressed in chondrocytes and transcriptomics performed to identify targets. miR-140-3p.1 and miR-140-3p.2 significantly down-regulated 694 and 238 genes, respectively, of which only 162 genes were commonly down-regulated. IsomiR targets were validated using 3′UTR luciferase assays. miR-140-3p.1 targets were enriched within up-regulated genes in rib chondrocytes of *Mir140*-null mice and within down-regulated genes during human chondrogenesis. Finally, through imputing the expression of miR-140 from the expression of the host gene *WWP2* in 124 previously published data sets, an inverse correlation with miR-140-3p.1 predicted targets was identified. Together these data suggest the novel seed containing isomiR miR-140-3p.1 is more functional than original consensus miR-140-3p seed containing isomiR.

## INTRODUCTION

MicroRNAs (miRNAs) are small noncoding RNAs that regulate gene expression. Mature miRNAs are processed from primary transcripts containing stem loops by cleavage mediated by Drosha and then Dicer proteins (Bartel 2004). The 5′ and 3′ sides of the stem loop can both give rise to mature miRNAs termed “-5p” and “-3p,” respectively (Griffiths-Jones 2004). These mature miRNAs are then loaded into the RNA-induced silencing complex (RISC) to mediate either mRNA degradation or translation inhibition of target mRNA through binding of the miRNA seed sequence (5′ bases 2–8) to the target 3′UTR (Bartel 2009). The level of complementarity between the seed and target mRNA is important for target repression. Binding to positions 2–7 of the miRNA may indicate target repression; however, either an adenine binding to position 1 of the miRNA (7a1 targets), a match at position 8 (7m8 targets) or both (8mer targets) can improve target repression (Lewis et al. 2005).

In recent years, small RNA (sRNA) sequencing (sRNA-seq) has identified additional miRNAs that do not perfectly align to the annotated mature miRNA, known as isomiRs (Morin et al. 2008). IsomiRs may be shorter or longer at the 5′ and/or 3′ end of the mature miRNA, and are not always templated to the genomic DNA. Variants at the 3′ end are quite common, although not thought to alter the miRNA target repertoire (Wyman et al. 2011). Variations at the 5′ end are less common but are of greater importance due to altered seed sequence, target repertoire and potentially the function of miRNAs. The type of 5′ isomiR variation is critical for determining the consequence on target repertoire, for example 5′ isomiRs of microRNAs can have highly overlapping (Cloonan et al. 2011; Llorens et al. 2013) or divergent (Tan et al. 2014) targets, which is dependent upon the presence or absence of a uracil (U) at position 2 of the longer sequence (Manzano et al. 2015).

The most studied miRNA in cartilage is miR-140, which produces miR-140-5p and miR-140-3p (Wienholds et al. 2005; Miyaki et al. 2009, 2010). Deletion of *Mir140* in mice leads to a skeletal phenotype including an osteoarthritis (OA)-like disease. Although the contribution of the lack of miR-140-5p or miR-140-3p to the phenotype remains largely undetermined, the majority of studies have focused on miR-140-5p rather than miR-140-3p (Wienholds et al. 2005; Miyaki et al. 2009, 2010), with the reason behind this -5p bias unclear. Barter et al. (2015) showed a particularly important role for miR-140-5p during human chondrogenesis with many genes under its control, and although they used a systematic approach, they did not include isomiRs within their analysis. miR-140-3p is less well studied than miR-140-5p and only a small number of -3p targets have been identified, which so far appear to have little relevance to cartilage and OA. Intriguingly, using sRNA-seq of human cartilage RNA we identified that miR-140-3p was high in abundance (>10-fold) compared to miR-140-5p (Crowe et al. 2016).

Although there have been several studies to elucidate the role of miRNAs in cartilage and chondrogenesis, the role of isomiRs has been largely ignored. Here we show miR-140-3p isomiRs are abundantly expressed in cartilage and that these isomiRs are present in RISC. Using over expression followed by transcriptomic analysis we show two miR-140-3p isomiRs (miR-140-3p.1 and miR-140-3p.2) have largely discrete target repertoires. We validated a number of common and discrete targets for each isomiR using a luciferase reporter system. We present evidence that miR-140-3p.1, which is not currently annotated in miRBase, may play roles in human chondrogenesis, mouse cartilage and in multiple other skeletal tissues.

## RESULTS

### RNA-seq of articular cartilage identifies an overabundance of miR-140-3p and miR-140-3p isomiRs

We performed sRNA-seq of human cartilage RNA and identified novel cartilage specific miRNAs (Crowe et al. 2016). Our cartilage sRNA-seq aligned to 990 miRNAs annotated in miRBase (Kozomara and Griffiths-Jones 2011), 704 of which contained at least one additional type of potential isomiR (Supplemental Table S1). As 5′ isomiRs are most likely to alter function, we focused further sRNA-seq analysis on miRNAs with 5′ isomiRs. Using thresholds of >100 reads and >5% of total reads for that miRNA, we identified 5′ isomiR sequences for 29 miRNAs, 26 of which have a single addition (includes templated and nontemplated additions) or deletion at the 5′ nt, while miR-3074-5p, miR-455-3p, and let-7b-3p all have two additional 5′ nt (Supplemental Fig. S1). Although the isomiR for miR-1246 is just under our read threshold (read count of 99), it appears to have four additional 5′ nt compared to the miRBase annotation (Supplemental Fig. S1).

**FIGURE 1.**
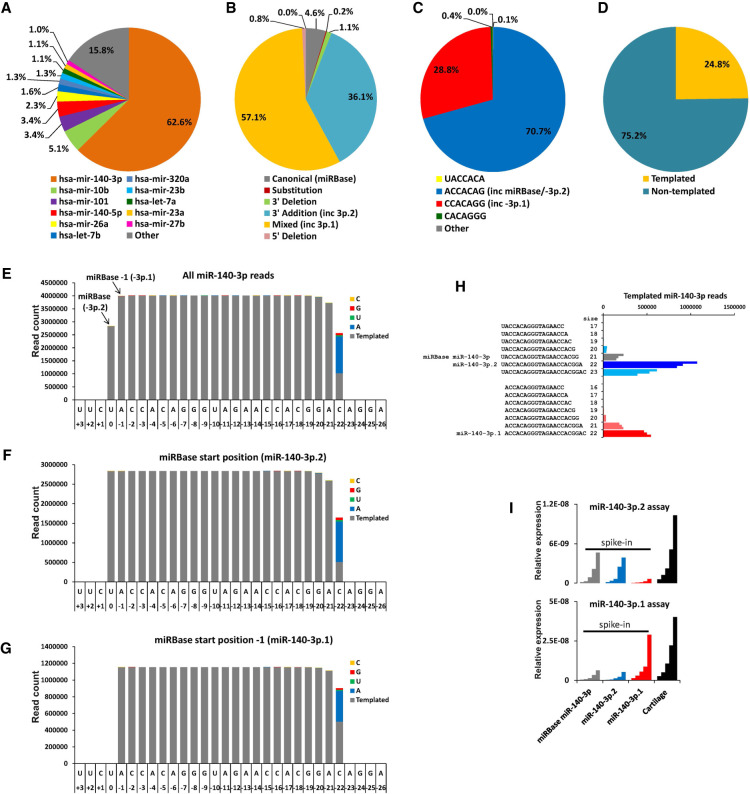
miR-140-3p isomiRs are abundantly expressed in cartilage. (*A*) Pie chart illustrating the relative abundance of all miRNAs expressed in cartilage, data combines isomiR and canonical reads for each miRNA. (*B*) Pie chart illustrating the isomiR type for all reads aligned to miR-140-3p. (*C*) Pie chart illustrating seed sequences for all reads aligned to miR-140-3p, >99% of reads have one of two seed sequences; ACCACAG (blue) and CCACAGG (red). (*D*) Pie chart of the percentage of templated and nontemplated reads for miR-140-3p within cartilage. (*E*) Histogram of all sequencing reads aligned to miR-140-3p in cartilage. (*F*) Histogram of sequencing reads aligned to miR-140-3p, whose 5′ end is as indicated in miRBase. (*G*) Histogram of sequencing reads aligned to miR-140-3p, whose 5′ end is one nucleotide shorter than indicated in miRBase. (*H*) Bar chart indicting the sequences and number of reads that contribute to each of the two seed sequences. Individual bars represent total read number from three separate individuals. (*I*) qRT-PCR for miR-140-3p.1 and miR-140-3p.2. Assays designed to detect each isomiR are able to distinguish between miR-140-3p.1 and miR-140-3p.2 spike-ins (twofold serial dilution) and detect high expression of each isomiR in cartilage (twofold serial dilution).

miR-140-3p and its isomiRs account for more than half of all sequencing reads in our cartilage sRNA-seq (Fig. 1A). As miR-140 is the most studied cartilage miRNA, yet most attention has been paid to miR-140-5p, this report will focus on mirR-140-3p and its isomiRs. IsomiRs can be subdivided into several categories, essentially either templated or nontemplated with either 5′ or 3′ modifications (Cloonan et al. 2011). miR-140-3p isomiRs are predominantly 3′ additions and mixed type isomiRs, with only 5% aligning to the original miRBase annotation (Fig. 1B). More than 99% of all miR-140-3p isomiRs result in one of two seed sequences; ACCACAG as annotated in miRBase, and CCACAGG, which is shifted by −1 nt (shifted by one nucleotide in the 3′ direction) (Fig. 1C). Twenty-five percent of reads for miR-140-3p were perfectly templated to DNA (Fig. 1D), the remainder contained one or more nontemplated nucleotides, predominantly nontemplated 3′ adenine additions (Fig. 1E), which were observed in sequencing reads containing both of the seed sequences (Fig. 1F,G). The most detected isomiRs with each seed are termed miR-140-3p.1 (ACCACAGGGTAGAACCACGGAC, seed CCACAGG) and miR-140-3p.2 (TACCACAGGGTAGAACCACGGA seed: ACCACAG), respectively (Fig. 1H). Expression of miR-140-3p.1 and miR-140-3p.2 in cartilage (performed with a twofold serial dilution) was validated using qRT-PCR with isomiR selective assays (Fig. 1I).

Analysis of other published sRNA-seq data identified the presence of miR-140-3p.1 and miR-140-3p.2 in human tissue types, although expressed at a lower level than in cartilage (Supplemental Fig. S2; Kuchen et al. 2010; Stark et al. 2010; Witten et al. 2010). As miRNA bound to Argonaute (AGO) indicates the ability to repress targets (Flores et al. 2014), we analyzed sRNA-seq data following immunoprecipitation of AGO proteins (Burroughs et al. 2011). Indeed, both miR-140-3p.1 and miR-140-3p.2 were present (Supplemental Fig. S2). Furthermore analysis of CLEAR-CLIP, a modified version of high-throughput sequencing of RNA isolated by crosslinking immunoprecipitation (HITS-CLIP), from mouse brain (Moore et al. 2015), identified sequences corresponding to both miR-140-3p.1 and miR-140-3p.2 ligated to endogenous mRNA potential targets (Supplemental Fig. S2). Together these data indicate miR-140-3p.1 and miR-140-3p.2 can be loaded into RISC and can target transcripts.

### miR-140-3p.1 and miR-140-3p.2 are predicted to have distinct targets

**FIGURE 2.**
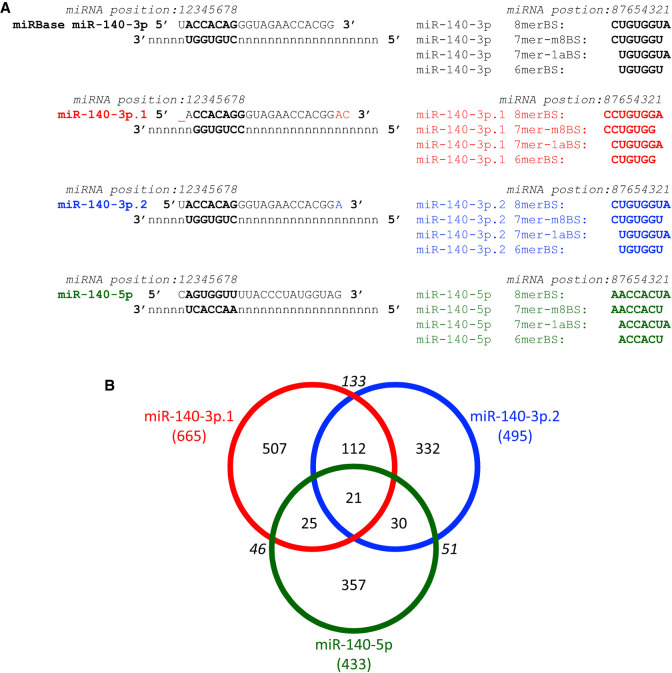
miR-140-3p.1 and miR-140-3p.2 are predicted to have distinct targets. (*A*) Schematic illustrating the sequences of miRBase miR-140-3p, miR-140-3p.1, miR-140-3p.2, and miR-140-5p and their potential binding sites within target mRNAs using the TargetScan categorization. (*B*) Venn diagram illustrating little cross over of targets predicted by TargetScan 7.2 for miR-140-3p.1, miR-140-3p.2, and miR-140-5p.

miRNAs generally target mRNAs through interaction of their seed sequence (nucleotides 2–7); the seed sequences for miR-140-3p.1 and miR-140-3p.2 differ (CCACAGG and ACCACAG, respectively; Fig. 2A). Thus, each miR-140-3p isomiR has different preferences for seed binding sites (Fig. 2A). Indeed, target prediction analysis for miR-140-3p.1 and miR-140-3p.2 by TargetScan 7.2 (conserved) identifies only 133 shared targets, a small number compared to the total number of predicted targets (665 and 495 for miR-140-3p.1 and miR-140-3p.2, respectively, Fig. 2B; Supplemental Table S2). The majority of these targets also differ from predicted miR-140-5p targets (Fig. 2B; Supplemental Table S2).

### Identification and validation of miR-140-3p.1 and miR-140-3p.2 targets in human chondrocytes

**FIGURE 3.**
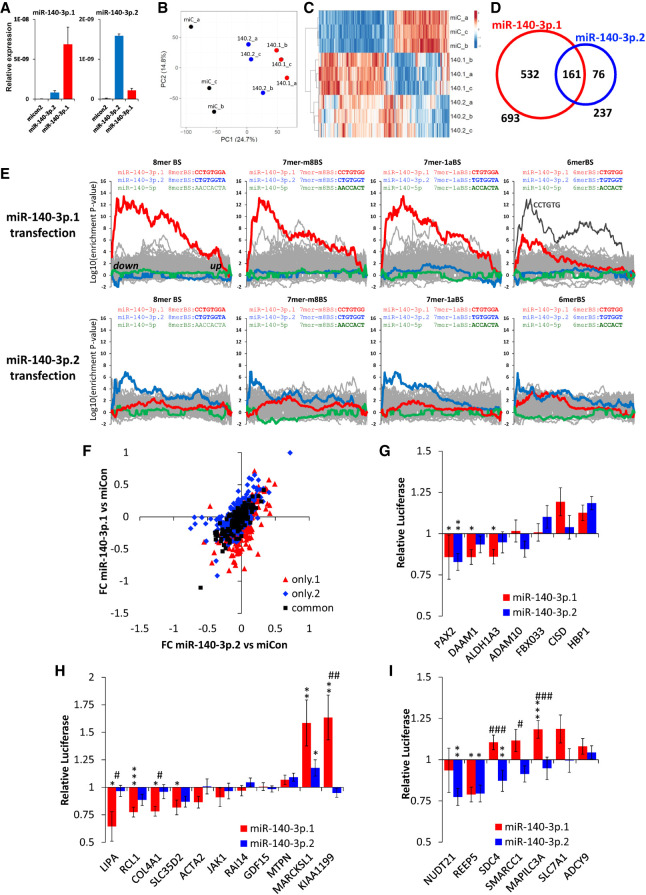
Identification and validation of miR-140-3p.1 and miR-140-3p.2 targets in human chondrocytes. (*A*) qRT-PCR for miR-140-3p.1 and miR-140-3p.2 following transfection of either control mimic, miR-140-3p.1 mimic or miR-140-3p.2 mimic for 48 h. (*B*) PCA of whole genome micro-array following transfection of mimics described in (*A*), (140.1 = miR-140-3p.1 and 140.2 = miR-140-3p.2). (*C*) Heat map of whole genome micro-array following transfection of mimics as described in (*A*) (140.1 = miR-140-3p.1 and 140.2 = miR-140-3p.2). (*D*) Venn diagram indicating the number of genes down-regulated following overexpression of miR-140-3p.1 and miR-140-3p.2. (*E*) Enrichment analysis using Sylamer for 3′UTR mRNA motifs complementary to either miR-140-3p.1 or miR-140-3p.2 seed sequences in genes whose expression decreased following transfection of either miR-140-3p mimic, miR-140-3p.1 mimic, or miR-140-3p.2 mimic. (*F*) Gene expression changes of miR-140-3p.1 (only.1; red triangles), miR-140-3p.2 (only.2; blue diamonds), or miR-140-3p.1 and miR-140-3p.2 (common; black squares) predicted targets following transfection of either miR-140-3p.1 mimic or miR-140-3p.2 mimic. (*G*–*I*) 3′UTR luciferase reporters for both miR-140-3p.1 and miR-140-3p.2 (*G*), miR-140-3p.1 (*H*), or miR-140-3p.2 (*I*) putative targets. (*), (**), or (***) indicate level of significance (*P* < 0.05, 0.01, or 0.001, respectively) of either miR-140-3p.1 or miR-140-3p.2 to control. # indicates level of significance (*P* < 0.05, 0.01, or 0.001, respectively) between miR-140-3p.1 and miR-140-3p.2.

Next, we used overexpression of miR-140-3p.1 and miR-140-3p.2 in primary human chondrocytes followed by genome-wide transcriptomics to experimentally identify similarities and differences in their target repertoire. Overexpression of miR-140-3p.1 and miR-140-3p.2 was validated using isomiR-specific qRT-PCR (Fig. 3A). Principal component analysis (PCA) and heat map illustrated clustering of biological replicates and differences between miR-140-3p.1 and miR-140-3p.2 in transfected chondrocytes (Fig. 3B,C). 693 and 237 genes were significantly (adjusted *P*-value<0.05) down-regulated by miR-140-3p.1 and miR-140-3p.2, respectively; however, only 161 genes were commonly down-regulated by both isomiRs (compared to control; Fig. 3D; Supplemental Tables S3, S4). DAVID pathway analysis of repressed genes identified enrichment terms associated with “phosphoprotein” for miR-140-3p.1 (Supplemental Table S5). Direct comparison of miR-140-3p.1 transfected with miR-140-3p.2 transfected cells revealed 237 genes were significantly different between the two isomiRs (Supplemental Tables S3, S4). DAVID pathway analysis of the 237 genes differentially expressed between the two isomiRs identified an enrichment of triple helical collagen genes (*COL2A1, COL4A1, COL6A3, COL11A1, COL11A2*; *P* = 0.0004, adjusted *P*-value = 0.066; Supplemental Table S5). Similarly, g-Profiler analysis of genes differentially expressed between miR-140-3p.1 and miR-140-3p.2 transfected chondrocytes, revealed an enrichment of genes associated with ECM structure including “connective tissue development” (adjusted *P*-value 0.005; Supplemental Fig. S3). The fraction of these genes that are decreased by, and are predicted to be a target of each isomiR, is greater for miR-140-3p.1 than for miR-140-3p.2, suggesting miR-140-3p.1 directly regulates genes belonging to these terms. However, the majority of genes contributing to the enrichment are not predicted direct targets of either miR-140-3p.1 or miR-140-3p.2, suggesting miR-140-3p isomiRs, in the most part, regulate these processes through indirect mechanisms. Together these data indicate that the sequence differences between the two isomiRs may be functionally important.

Sylamer, an unbiased motif analysis tool, was used to investigate miRNA binding site enrichment within down-regulated 3′UTRs (van Dongen et al. 2008). Transfection of either miR-140-3p.1 or miR-140-3p.2, showed specific enrichment for 8mer, 7m8, 7a1, and 6mer seed binding sites of the transfected isomiR. Interestingly, a greater enrichment was observed for miR-140-3p.1 (noncanonical seed) binding sites than miR-140-3p.2 (canonical seed) binding sites (Fig. 3E). Predicted targets (identified using TargetScan 7.2) that are unique to either miR-140-3p.1 or miR-140-3p.2 were down-regulated following overexpression of either miR-140-3p.1 or miR-140-3p.2, respectively, but not down-regulated by the reciprocal isomiR (Fig. 3F), suggesting both isomiRs can repress specific transcripts in human chondrocytes.

Based upon expression of individual genes (Supplemental Table S3), unique and common targets were then selected for 3′UTR luciferase validation (Fig. 3G–I). As expected *LIPA*, *RCL1*, *COL4A1*, and *SLC35D2* were validated as miR-140-3p.1 targets; *NUDT21*, *REEP5*, and *SDC4* were validated as miR-140-3p.2 targets and *PAX2* validated as a target of both miR-140-3p.1 and miR-140-3p.2. Interestingly, the *REEP5* 3′UTR luciferase construct was also repressed by miR-140-3p.1, although not a predicted target of miR-140-3p.1. Conversely, *DAAM1* and *ALDH1A3* are predicted targets of both miR-140-3p.1 and miR-140-3p.2; however, significant repression was only observed for miR-140-3p.1 and not for miR-140-3p.2. Contrary to the repression role of miRNAs in gene regulation, miR-140-3p.1 increased luciferase for the *MARCKSL1*, *KIAA1199*, and *MAPILC3A* 3′UTR reporters.

### miR-140-3p isomiRs are functional in mouse cartilage

**FIGURE 4.**
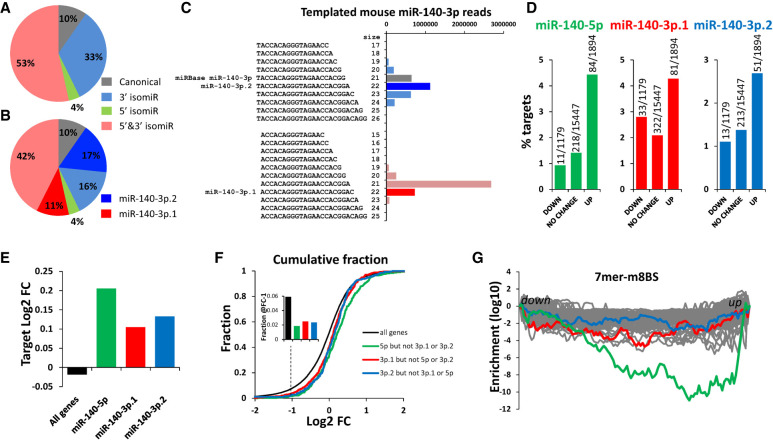
Evidence for functional miR-140-3p.1 and miR-140-3p.2 in mouse cartilage. (*A*) Pie chart illustrating the fraction of miR-140-3p reads with canonical 5′ and 3′ ends (gray), canonical 5′ and either shorter or longer 3′ (blue), canonical 3′ and either shorter or longer 5′ (green), either shorter or longer 5′, and either shorter or longer 3′ (red). (*B*) As in (*A*) with miR-140-3p.1 and miR-140-3p.2 indicated in dark red and dark blue, respectively. (*C*) Bar chart indicting number of reads that contribute to (*B*). (*D*) % of genes that are predicted targets of either a miR-140-5p (green), miR-140-3p.1 (red), or miR-140-3p.2 (blue), in genes whose expression decrease, does not change or increase following KO of miR-140 in mice. (*E*) Median fold change (FC) of all genes that are predicted targets of either a miR-140-5p (green), miR-140-3p.1 (red), or miR-140-3p.2 (blue) following KO of miR-140 in mice. (*F*) Cumulative fraction plot of all genes (black), miR-140-5p (green), miR-140-3p.1 (red), or miR-140-3p.2 (blue) predicted targets, for genes ordered from most decreased expression to most increased expression in *Mir140*-null mice. (*Inset*) Fraction at a cut-off of log_2_FC-1. (*G*) Sylamer analysis for *Mir140*-null mice; the 100 most enriched 7mers are shown with 7m8 binding sites for miR-140-5p (green), miR-140-3p.1 (red), and miR-140-3p.2 (blue) highlighted.

Analysis of mouse sRNA-seq data revealed that both miR-140-3p.1 and miR-140-3p.2 are present (Fig. 4A–C) and more abundant than miR-140-5p (Supplemental Fig. S4A; Chiang et al. 2010), indicating miR-140-3p 5′ isomiRs are conserved across species, although there are some differences in abundance of 3′ variations. We and others have generated *Mir140*-null mice, which lack all miRNAs and isomiRs produced from the *Mir140* locus (Supplemental Fig. S5). Transcriptome RNA-seq data of rib cartilage from these mice identified 1894 and 1179 genes that were significantly up- and down-regulated, respectively (Supplemental Tables S6, S7). Pathway analysis of up-regulated genes following knock out (KO) of *Mir140* identified terms associated with “phosphoprotein” (Supplemental Table S8), which is consistent with pathway analysis of genes decreased following miR-140-3p.1 overexpression in human chondrocytes (Supplemental Table S5). miR-140-5p, miR-140-3p.1, and miR-140-3p.2 predicted targets (identified using TargetScan 7.2) were enriched within up-regulated genes (Fig. 4D), and their average expression increased following KO of the *Mir140* locus (Fig. 4E), indicating a loss of target repression. Furthermore, cumulative fraction plot analysis indicates that the fraction of targets that decrease following KO of the *Mir140* locus is lower than expected (Fig. 4F). Unbiased Sylamer analysis also identified an enrichment of miR-140-5p, miR-140-3p.1, and miR-140-3p.2 7m8 seed binding sites within up-regulated genes; miR-140-5p displayed the greatest enrichment, followed by miR-140-3p.1 and then miR-140-3p.2 (Fig. 4G).

More than 99% of miRNAs produced from the murine *Mir140* locus share seed binding sites with either miR-140-5p, miR-140-3p.1, or miR-140-3p.2; of these miR-140-5p has the lowest read count, yet has the largest contribution to target repression (Fig. 4). We used Sylamer to investigate the possibility that other lowly expressed sequences produced from the *Mir140* stem loop (Supplemental Fig. S4A), may contribute to target repression. This analysis indicates miR-140-5p followed by miR-140-3p.1 have the largest contribution to target repression in mir140 KO mice (Supplemental Fig. S4B), and that there are no lowly expressed *Mir140*-derived miRNAs that have a major contribution to target repression in mice.

### miR-140-3p.1 targets are enriched within down-regulated genes during MSC chondrogenesis

**FIGURE 5.**
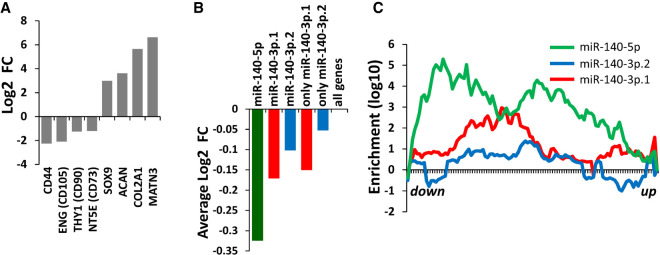
Evidence for miR-140-3p.1 and miR-140-3p.2 function during human MSC chondrogenesis. Re-analysis of data from Barter et al. for human MSC differentiation to cartilage. (*A*) Changes in gene expression for eight MSC and chondrogenic markers. (*B*) Mean fold change of all genes that are predicted targets of either a miR-140-5p (green), miR-140-3p.1 (red), or miR-140-3p.2 (blue) during MSC chondrogenesis. (*C*) Sylamer analysis for a miR-140-5p (green), miR-140-3p.1 (red), and miR-140-3p.2 (blue) 7m8 seed sequences.

miR-140 is highly up-regulated during mesenchymal stem cell (MSC) chondrogenesis, with miR-140-5p predicted targets highly enriched within the down-regulated genes (Barter et al. 2015). Here we show through reanalysis of this transcriptomic data set (Fig. 5A) that expression of predicted targets of both miR-140-3p.1 and miR-140-3p.2 are decreased during chondrogenesis. Similar to miR-140-5p predicted targets, the average expression of miR-140-3p.1 and miR-140-3p.2 predicted targets decreased during chondrogenesis, with unique miR-140-3p.1 targets being more repressed than unique miR-140-3p.2 predicted targets (*P* = 0.07) (Fig. 5B). Sylamer analysis indicated enrichment for miR-140-5p and miR-140-3p.1, but minimal enrichment for miR-140-3p.2 (Fig. 5C). Consistent with *Mir140*-null mouse data, the enrichment of miR-140-5p was greater than for both miR-140-3p.1 and miR-140-3p.2 in genes whose expression decreased during human MSC chondrogenesis (Fig. 5C).

### miR-140-5p and miR-140-3p.1 predicted targets inversely correlate with *WWP2* in multiple skeletal tissues

Having established miR-140-5p, miR-140-3p.1, and miR-140-3p.2 targets are regulated during human MSC chondrogenesis and in mouse rib chondrocyte development, we next looked for evidence of an inverse correlation between miR-140 and target gene expression in other skeletal tissues. Whole genome transcriptomic data sets of skeletal tissues following various perturbations have been collated within SkeletalVis, a data portal for cross-species skeletal transcriptomics data (Soul et al. 2018). Although the majority of these studies do not directly assess miRNA expression, miRNAs that are located in the introns of protein coding genes, including miR-140, are frequently coregulated with their host gene (Franca et al. 2016). A number of studies have utilized host-gene expression to predict the gene targets of intronic miRNAs through a consistent negative correlation in expression (Gennarino et al. 2009; Radfar et al. 2011). miR-140 is located in intron 16 of *WWP2*, with expression of the miR and an abundant isoform of *WWP2* (transcript variant 2) controlled by a common promoter (Yamashita et al. 2012). *WWP2* was therefore used as a surrogate for miR-140 expression.

**FIGURE 6.**
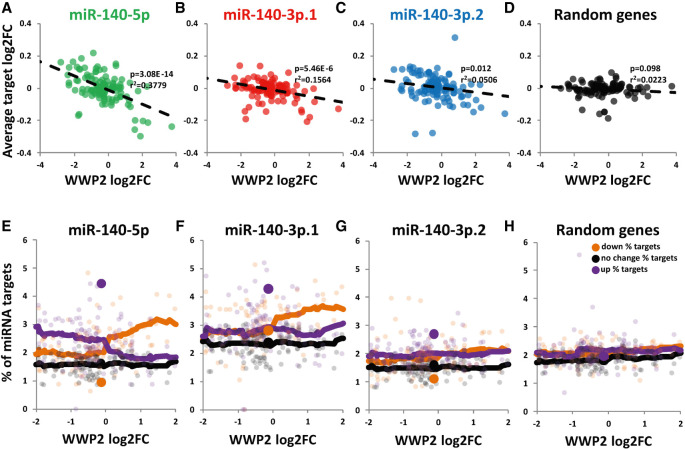
Correlation of *MIR140* locus host gene *WWP2* with miR-140 target genes in SkeletalVis transcriptomic data sets. (*A*–*D*) Correlation analysis of *WWP2* and predicted miRNA target expression in 124 preexisting transcriptome-wide comparisons (both human and mouse). Scatter graph of *WWP2* log_2_FC plotted against the average log_2_FC for genes predicted to be either a target of miR-140-5p (*A*), miR-140-3p.1 (*B*), miR-140-3p.2 (*C*), or 500 random genes (*D*), *P*-value calculated from linear regression analysis. (*E*–*H*) Percentage of miR-140-5p (*E*), miR-140-3p.1 (*F*) or miR-140-3p.2 (*G*) predicted targets and 500 random genes (*H*) within the significantly up- or down-regulated genes for data sets where *WWP2* expression significantly changes. The percentage of targets within up-regulated (purple), no change (black), or down-regulated (orange) transcripts is plotted against log_2_FC *WWP2*. Large icons represent enrichment of miR-140-5p, miR-140-3p.1, and miR-140-3p.2 predicted targets in up- and down-regulated genes within rib cartilage of *Mir140*-null mice, where *Wwp2* expression does not change.

Of the 779 skeletal gene expression responses within SkeletalVis, 124 contained a significant alteration (adjusted *P*-value <0.05, no fold change cutoff) in *WWP2* expression. The change in *WWP2* expression (as a surrogate for miR-140 expression) was plotted against the average fold change of predicted miRNA isomiR targets for each study. Using regression analysis we identified a significant inverse relationship between *WWP2* and miR-140-5p (Fig. 6A; *r*^2^ = 0.38, *P* = 3.08 × 10^−14^) and miR-140-3p.1, predicted targets (Fig. 6B; *r*^2^ = 0.16, *P* = 5.46 × 10^−6^). There was a minimal inverse relationship between *WWP2* and miR-140-3p.2 predicted targets (Fig. 6C; *r*^2^ = 0.05, *P* = 0.012) or randomly selected genes (Fig. 6D). We then divided the studies into two; those where *WWP2* significantly decreased and those where *WWP2* significantly increased. The average target log_2_FC of predicted miR-140-5p, miR-140-3p.1, and miR-140-3p.2 targets was significantly higher in studies where *WWP2* decreased than studies where *WWP2* increased (Supplemental Fig. S6A). To determine if miR-140-5p, miR-140-3p.1, and miR-140-3p.2 targets are regulated in a similar way in each study, we correlated log_2_FC for miR-140-5p, miR-140-3p.1, and miR-140-3p.2 targets for all studies (Supplemental Fig. S6B). Average log_2_FC for miR-140-5p targets correlated most strongly with average log_2_FC for miR-140-3p.1 targets (Supplemental Fig. S6B). The direction of target FC tends to be in the opposite direction to *WWP2* FC (Supplemental Fig. S6B). Thus, where *WWP2* expression changes the expression of predicted miR-140 targets have a tendency to change in the opposite direction.

In addition to average log_2_FC of predicted targets, we also investigated the percentage of predicted targets that increased or decreased within each of the studies. Within studies where *WWP2* expression increased there was generally a larger percentage of predicted miR-140-5p and miR-140-3p.1 targets within down-regulated genes than within up-regulated genes; this was not true for miR-140-3p.2 (Fig. 6E–H). For the contrary, when *WWP2* expression decreased, there was generally a larger percentage of miR-140-5p targets within up-regulated genes than within down-regulated genes; however, there was little evidence of this for miR-140-3p.1 or miR-140-3p.2 (Fig. 6E–H). Within *Mir140*-null mice (large symbols), where *WWP2* expression was not altered, the percentage of targets within the up-regulated genes is greater than the percentage of targets within down-regulated genes (Fig. 6E–H).

Next we calculated “Target Enrichment (up/down)” by dividing the percentage of targets within up-regulated genes by those within the down-regulated genes, for each of the studies (a value >1 indicates more targets within up-regulated whereas a value <1 indicates more targets within down-regulated genes). Target enrichment for miR-140-5p and miR-140-3p.1 was significantly higher in studies where *WWP2* decreased than in studies where *WWP2* increased (Supplemental Fig. S6C). Similar to the log_2_FC analysis, the enrichment for miR-140-5p targets correlated most strongly with enrichment for miR-140-3p.1 targets (Supplemental Fig. S6D).

These data indicate that when *WWP2* expression increases, both miR-140-5p and miR-140-3p.1 predicted targets are repressed. Furthermore, they also indicate that miR-140-3p.2, which contains the miRBase annotated seed sequence, has a less significant biological effect on gene expression than either miR-140-5p or miR-140-3p.1.

To further refine prediction of putative direct miR-140 targets, we investigated the overlap between four criteria; TargetScan predicted targets, genes down-regulated following overexpression in human chondrocytes, genes up-regulated in mice lacking the *Mir140* locus, and genes up-regulated during MSC chondrogenesis. There were 3, 3, and 0 genes matching all of these criteria for miR-140-5p, miR-140-3p.1, and miR-140-3p.2, respectively (Supplemental Fig. S7A; Supplemental Table S9). *ABCA1* was common to both miR-140-5p and miR-140-3p.1. Furthermore, *ABCA1* was inversely correlated with *WWP2* across multiple skeletal data sets, with the inverse correlation being strongest in cartilage experiments (Supplemental Fig. S7B), where miR-140 expression is highest.

## DISCUSSION

IsomiRs of miR-140-3p have previously been detected in chondrocytes (Haseeb et al. 2017), breast cancer cell lines (Salem et al. 2016), and endometrial tissue (Krawczynski et al. 2015), and are now recognized in TargetScan 7.2 (Agarwal et al. 2015b). In TargetScan and this study, miR-140-3p.2 refers to the miRNA sequences with the seed of the original miRBase annotation and miR-140-3p.1 refers to the miRNA sequences with the seed of the newly identified isomiR. This study focuses upon the impact of seed sequence (5′ isomiRs); further investigation is therefore required to understand the role of 3′ isomiRs of miR-140-3p. miRBase does not currently include specific annotation for isomiRs. In addition to miR-140-3p isomiRs, we detected isomiRs for a number of other miRNAs in cartilage, including miR-455, which is also now recognized in TargetScan 7.2 (Agarwal et al. 2015b). Sequence analysis of the miR-1246 isomiR, suggests it may in fact be miR-1290. According to miRBase these two miRNAs are transcribed from two separate loci on chromosome 2 and 1, respectively. However, Mazieres et al. (2013), suggest miR-1246 and miR-1290 are processed from a common transcript (*RNU2*), which explains their similar sequence and expression pattern during chondrogenesis (Barter et al. 2015). Many of the other detected cartilage isomiRs can be transcribed from more than one genomic location (hsa-mir-199a-3p, hsa-mir-199b-3p, hsa-mir-29b, hsa-mir-101, hsa-mir-320c, hsa-mir-103b), raising the possibility that each genomic location gives rise to only one mature miRNA, which are then perceived as isomiRs.

Primary miRNA transcripts are processed to pre-miRNAs in the nucleus by DROSHA (a dsRNA nuclear type III endoribonuclease) facilitated by DGCR8, and subsequently cleaved in the cytoplasm by DICER to generate functionally mature miRNAs molecules. IsomiRs are generated because both DICER and DROSHA process miRNA precursors imprecisely generating miRNA variants with several plus/minus nucleotides at the 5′ and/or 3′ (Neilsen et al. 2012). In addition, miRNAs can also be posttranscriptionally adenylated or uridylated, which could modify miRNA targeting properties and/or their stability (Burroughs et al. 2010; Marzi et al. 2016). The importance of endogenously expressed 5′ isomiRs has been demonstrated by Chiang et al. (2010), where they showed deletion of miR-223 in mouse neutrophils (Baek et al. 2008), resulted in an increase in expression of predicted targets of a lesser expressed 5′ isomiR of the microRNA. Although it is established that 5′ isomiRs can regulate gene expression, the functional reason for their presence is still debated. 5′ isomiRs of a given microRNA can have highly overlapping targets, potentially increasing the specificity of regulation of particular pathways (Cloonan et al. 2011; Llorens et al. 2013). However, 5′ isomiRs can also increase a microRNA's target repertoire (Tan et al. 2014). Manzano et al. (2015) suggest these apparent discrepancies are dependent upon the presence or absence of a U at position 2 of the longer isomiR sequence. miR-140-3p.2, the longest 5′ isomiR of miR-140-3p, does not possess a U at position 2, which would suggest miR-140-3p.1 and miR-140-3p.2 have divergent target repertoires, decreasing their effect on any single target (Manzano et al. 2015). Although not directly studied here, 3′ isomiRs are widespread, with 3′ adenine additions being most common and reported to decrease the targeting ability of the miRNA (Burroughs et al. 2010). Some of the miR-140-3p isomiRs with the same seeds as miR-140-3p.1 and miR-140-3p.2 have 3′ adenine additions, which in combination with divergent 5′ isomiRs may account for the poor target repression by miR-140-3p.1 and miR-140-3p.2 compared with miR-140-5p. Interestingly, there is an inverse correlation between the ability to repress targets (miR-140-5p > miR-140-3p.1 > miR-140-3p.2) and expression in cartilage (miR-140-3p.2 > miR-140-3p.2 > miR-140-5p).

A mutation within miR-140-5p has recently been identified as causing a human skeletal dysplasia (Grigelioniene et al. 2019b). Mice with the corresponding miR-140-5p mutation phenocopy the human situation and display a loss of miR-140-5p target repression (in rib chondrocytes). This is thought to contribute to the phenotype since the expression of miR-140-3p.1 and miR-140-3.2 were not affected by the mutation. Interestingly though, the phenotypes of the miR-140-5p mutant mice and *MIR140*-null mice show some differences, which could perhaps be accounted for by the latter animals lacking miR-140-3p and the isomiRs described here.

Here we identify discrete targets for miR-140-3p.1 and miR-140-3p.2. LIPA, a cholesterol ester hydrolase, is a target of miR-140-3p.1 but not miR-140-3p.2. *LIPA* expression decreases during chondrocyte development in humans (Wu et al. 2013), consistent with high miR-140-3p.1 expression in chondrocytes. SDC4 (Syndecan-4), a target of miR-140-3p.2 but not miR-140-3p.1, has been implicated in osteoarthritis progression through activation of ADAMTS5 (Echtermeyer et al. 2009), suggesting *SDC4* suppression by miR-140-3p.2 may maintain a healthy cartilage phenotype. *HMGCR* and *HMGCS1* have now been identified as targets of miR-140-3p.1 in breast cancer cells (Bhardwaj et al. 2018); however, their expression was not repressed by miR-140-3p.1 in human chondrocytes, indicating miR-140 isomiRs act in a tissue-specific manner. Even where repression was determined, the effects of miR-140-3p.1 and miR-140-3p.2 were relatively small (up to ∼25% repression) when compared to the effects of miR-140-5p on a previously published miR-140-5p direct target, FZD6 (∼60% repression) (Barter et al. 2015). Furthermore, contrary to the expected miR-140-3p.1 repression of targets, miR-140-3p.1 increased luciferase for the *MARCKSL1*, *KIAA1199*, and *MAPILC3A* 3′UTR constructs, possibly suggesting stabilization of transcript, as previously described for miR-322 and *MEK1* (Bluhm et al. 2017).

The target repertoire of miR-140-3p.1 and miR-140-3p.2 are vastly different, due to established miRNA target interaction rules. The only shared binding motif is “CUGUGG,” which can be recognized by miR-140-3p.1 as a 6merBS (CUGUGG), or by miR-140-3p.2 as a 7m8-BS or 8mer-BS if followed by U or UA, respectively (CUGUGG**U** or CUGUGG**UA**) (Fig. 2A). Indeed the miR-140-3p.1 6merBS (CUGUGG) was the most enriched miR-140-3p.1 motif following miR-140-3p.2 transfection (Fig. 3E; red line in bottom right), indicating miR-140-3p isomiRs act according to traditional miRNA targeting rules. Other types of miRNA binding sites such as 3′ compensatory binding (Grimson et al. 2007) may be shared between miR-140-3p.1 and miR-140-3p.2, but are less common and more difficult to predict.

Analysis of miRNA target interactions within published skeletal data sets, using the *WWP2* host gene as a surrogate for miRNA expression, along with directly overexpressing the miRNAs and observing changes in gene expression, suggests these types of analyses can improve miRNA target prediction within skeletal tissues. Caution must however be taken as *WWP2* and miR-140 can display tissue specific expression (Yamashita et al. 2012); furthermore, miRNA are often coexpressed with their targets as part of a larger more complex network (Cora et al. 2017). This type of coexpression may account for the studies where a correlation, rather than an inverse correlation, between *WWP2* and miR-140 targets was observed. Where an inverse correlation was observed between *WWP2* and miR-140 predicted targets this was greatest within cartilage. Together, these data indicate the miR-140-3p isomiR miR-140-3p.1 can function to down-regulate transcript expression in vitro and in vivo.

In conclusion, analysis of human cartilage sRNA-seq identified an abundant isomiR of miR-140-3p, miR-140-3p.1. Functional analysis in human and murine chondrocytes and analysis of published skeletal data sets suggests the newly identified isomiR is more effective than the original miR-140-3p annotated in miRBase. These data suggest the function of miR-140-3p, which has previously been largely ignored, should be revisited.

## MATERIALS AND METHODS

### Analysis of cartilage sRNA-seq

Cartilage sRNA-seq, from chondrocytes taken from three independent osteoarthritic knees, was analyzed as previously described (Sorefan et al. 2012; Crowe et al. 2016). cDNA libraries were generated from small RNA isolated from chondrocytes taken from three independent osteoarthritic knees. 5′ isomiRs were defined using the following criteria: (i) loss or gain of 1 or more nucleotides, using the mature miRBase (release 22.1) (Kozomara and Griffiths-Jones 2011) sequences as a reference; (ii) a read count >100; and (iii) a read count > 5% of the mature miRBase reference sequence for that miRNA.

### Analysis of noncartilage sRNA-seq

Read count for noncartilage sRNA-seq including; melanoma (Stark et al. 2010), cervix (Witten et al. 2010), lymphocytes (Kuchen et al. 2010) was obtained from miRBase (Kozomara and Griffiths-Jones 2011), or directly from publication for sRNA-seq following immunoprecipitation of Argonaute (AGO) proteins (Burroughs et al. 2011). Sequences were designated as either miR-140-3p.1 or miR-140-3p.2 based upon seed sequence. For the analysis, mouse cortex CLEAR (covalent ligation of endogenous Argonaute-bound RNAs)-CLIP data (NCBI GEO GSE73059) was analyzed as previously described (Moore et al. 2015) using the parameters provided. Briefly, the reads were quality filtered, duplicate reads collapsed, trimmed and the barcodes stripped using the CLIP Tool kit (Shah et al. 2017). The TargetScanMouse 7.2 miRNA database was mapped against each sample (treated as a reference genome) using Bowtie (v1.0.0). miRNA-first chimeric reads uniquely assignable to the miR-140 isomiRs were identified in R; potential PCR duplicates with the same degenerate barcode were collapsed and filtered to retain reads with chimeric mRNA sequences >20 nt in length.

### Human articular chondrocyte isolation, culture, and transfection

Human articular chondrocyte isolation from knee cartilage was performed as previously described (Barter et al. 2015). Tissue was donated by patients with diagnosed osteoarthritis and undergoing joint replacement surgery, with informed consent and ethics committee approval. Briefly, macroscopically normal cartilage was removed from the subchondral bone and dissected into ∼1 mm pieces using scalpel and forceps. Enzymatic digestion was performed using trypsin and then collagenase overnight at 37°C. Chondrocytes were then grown to confluence and plated into six well plates for miRNA/isomiR mimic transfection. An amount of 100 nM miRNA mimic and control were transfected into HAC using DharmaFECT 1 transfection reagent (Dharmacon, Horizon Discovery) according to the manufacturer's protocol and essentially as previously described (Barter et al. 2015). Forty-eight hours posttransfection HAC were lysed and RNA harvested using Qiagen miRNeasy kit (Qiagen). Custom miRNA mimics with each isomiR sequence were purchased from Dharmacon, standard (A4) desalted purification.

### Microarray and identification of targets in human chondrocytes

Microarray was performed by Cardiff University, Central Biotechnology Services, using Illumina whole-genome expression array Human HT-12 V4 (Illumina). Normalization of the quantified signals (background corrected) was performed using the global Lowess regression algorithm. Expression analysis was performed in R/bioconductor using the limma package (Smyth 2004). PCA and heat map were generated using ClustVis (Metsalu and Vilo 2015). PCA analysis was performed on normalized expression values of all unique genes detected by the microarray analysis. Data was not transformed; row scaling was unit variance and the PCA method selected was singular value decomposition (SVD) with computation. Heat map is based on the top 650 significant genes for each comparison (miC vs. mR-140-3p.1 or miC vs. miR-140-3p.2), which after removal of duplicates gave 882 genes in total. Clustered distance was based on correlation with the method set to average. TargetScanHuman (version 7.2 [Agarwal et al. 2015a]) was used to predict targets of miR-140-3p.1 and miR-140-3p.2. In order to demonstrate discrete functions we investigated unique targets for each isomiR (3p.1 and 5p targets were removed from 3p.2 analysis; 3p.1 and 5p targets were removed from 3p.1 analysis; 3p.1 and 3p.2 targets were removed from 5p analysis). Genes were selected for luciferase validation based on both altered expression in miR-140-3p.1 or miR-140-3p.2 transfected cells versus control and on expression in miR-140-3p.1 transfected cells versus expression in miR-140-3p.2 transfected cells.

### Target enrichment and pathway analysis

Sylamer analysis was performed on ordered gene lists from most down-regulated to most up-regulated (including genes whose expression did not significantly change) (van Dongen et al. 2008). Target enrichment (%targets) was calculated by dividing the total number of predicted targets (TargetScan 7.2) that significantly increased or decreased by the total number of genes that significantly increased or decreased, multiplied by 100. Cumulative fraction plots were generated as previously described (Woods et al. 2019), using ordered gene list from down-regulated to most up-regulated (regardless of significance) and predicted targets from TargetScan 7.2. Pathway analysis was performed using Database for Annotation, Visualization, and Integrated Discovery (DAVID) v6.7 (Huang da et al. 2009) and g-Profiler (Raudvere et al. 2019).

### Q-RT-PCR for miR-140-3p.1 and miR-140-3p.2

Custom miR-140-3p.1 and miR-140-3p.2 assays (Exiqon, Qiagen) were used to detect expression of miR-140-3p.1 and miR-140-3p.2. Assays were validated using spike-in of miR-140-3p.1 and miR-140-3p.2 mimic (Dharmacon).

### 3′UTR luciferase reporter construction and assay

In-Fusion cloning (Clontech, Takara Bio Europe SAS) of selected 3′UTRs into the pmirGLO vector (Promega) was used to generate 3′UTR luciferase reporters essentially as previously described (Supplemental Table S10; Barter et al. 2015). SW1353 chondrosarcoma cells were seeded and cultured to reach ∼50% confluence after 24 h (Barter et al. 2015). miRNA (100 nM) were transfected using DharmaFECT 1 transfection reagent; reporter plasmids (500 ng/mL) were transfected using FugeneHD (Promega). Twenty-four hours after transfection luciferase levels were determined using Promega dual luciferase assay and GloMax plate reader (Promega).

### Generation of miR-140^−/−^ mice

All animal experiments were performed under licenses granted from the Home Office (United Kingdom) in accordance with the guidelines and regulations for the care and use of laboratory animals outlined by the Animals (Scientific Procedures) Act 1986 according to Directive 2010/63/EU of the European Parliament, and conducted according to protocols approved by the Animal Ethics Committee of Newcastle University and the Home Office, United Kingdom. Postgeneration breeding and subsequent phenotyping were performed under licenses PPL60/4525 and P8A8B649A. CRISPR/Cas9 guide RNAs (crRNA) were designed using CHOPCHOP. crRNA linked with TRACR (sgRNA) were amplified by PCR with a pLKO vector (Addgene_52628) as template, using the primer T7 TRACR R (Supplemental Table S10) and a 5′ PCR primer that included a T7 sequence and the crRNA. This was converted to RNA using the MEGAshortscript T7 kit (Fisher Scientific). sgRNA (50 ng/mL each) were mixed with recombinant Cas9 (ToolGen, CamBioScience Limited) and injected into the cytoplasm of donor mouse zygotes and transferred into recipient foster mothers, all essentially as previously described (Ittner and Gotz 2007; Ran et al. 2013; Yang et al. 2013). The mixed C57BL/6 and CBA/ca F_0_ mice were backcrossed onto C57BL/6J and heterozygous animals crossed three times to eventually generate wild-type and null lines used in purification of rib chondrocytes. Genotype was confirmed by ear-notch PCR and Sanger sequencing (Supplemental Fig. S5).

### Mouse rib chondrocyte isolation and RNA-seq

Primary mouse costal chondrocytes were isolated from 7-d-old *Mir140^−/−^* and wild-type (WT) mice using collagenase digestion, essentially as previously described (Grigelioniene et al. 2019a). Total RNA, including miRNA was isolated using the miRVana miRNA Isolation Kit (with phenol) (Fisher Scientific). Sequencing libraries were prepared from 500 ng of purified total RNA using the Illumina TruSeq Stranded mRNA sample preparation kit according to the manufacturer's protocol, and sequenced on Illumina NextSeq500. Each sample provided >12 million single-end 75-bp sequencing reads. Sequenced reads were mapped to the mm10 transcriptome using Salmon (Patro et al. 2017). Batch effects were estimated using RUVseq (Risso et al. 2014) and incorporated into the differential expression analysis performed using DESeq2 (Love et al. 2014). PCA and heat map were generated using ClustVis (Metsalu and Vilo 2015) using the parameters defined above, using all transcripts for the PCA and the top 1200 significant transcripts based on *P*-value for the heatmap.

### Analysis of human MSC chondrogenesis

Microarray data of human MSC chondrogenesis was previously published by Barter et al. (2015). Data was reanalyzed for changes in expression of miR-140-3p.1 and miR-140-3p.2 predicted targets, lists of conserved predicted targets were obtained from TargetScan (version 7.2). Enrichment for 7m8 seed sequence binding sites was performed using Sylamer (van Dongen et al. 2008).

### SkeletalVis analysis

Gene expression responses within SkeletalVis were filtered for human and mouse data sets and where *WWP2* expression significantly changed (adjusted *P*-value is <0.05, no fold change cutoff), leaving 124 experimental comparisons. The change in *WWP2* expression was plotted against the average fold change of predicted (TargetScan7.2) miRNA targets for each experimental comparison. Correlation (*R*^2^) and regression analysis for all 124 experimental comparisons was calculated using the data analysis add-in in Microsoft Excel. The percentage (% of miRNA targets) was calculated by dividing the total number of predicted targets (TargetScan 7.2) that significantly increased or decreased by the total number of genes that significantly increased or decreased, multiplied by 100, for each of the 124 experimental comparisons. The percentage of miR-140-5p, miR-140-3p.1, and miR-140-3p.2predicted targets within up-, no change or down-regulated genes was then plotted against log_2_FC for *WWP2*; the trend lines represent the cumulative mean starting from studies where *WWP2* is most up- or down-regulated to studies where *WWP2* changes least, to demonstrate trend. A list of randomly generated genes was analyzed alongside miR-140-5p, miR-140-3p.1, and miR-140-3p.2. “Targets Enrichment (up/down)” was calculated by dividing the “% of miRNA targets within up-regulated genes” by the “% of miRNA targets within down-regulated genes,” this was performed for each miRNA in each study. The mean “Target enrichment (up/down)” for studies where *WWP2* either decreased or increased was plotted. “Targets Enrichment (up/down)” for miR-140-5p, miR-140-3p.1, and miR-140-3p.2 predicted targets was also plotted against each other. Experimental comparisons where *WWP2* decreased or increased were shown in different colors as described.

## DATA DEPOSITION

Microarray data and RNA-seq data are available at NCBI GEO data sets with the accession numbers GSE144374.

## SUPPLEMENTAL MATERIAL

Supplemental material is available for this article.

## Supplementary Material

Supplemental Material
