# Supplementary material for: microRNA-seq of cartilage reveals an overabundance of miR-140-3p which contains functional isomiRs
Source: RNA. 2020 Nov;26(11):1575–88. doi: 10.1261/rna.075176.120 (PMC7566571; doi:10.1261/rna.075176.120)
Supplement: Supplemental Material [file supp_26_11_1575__index.html]

microRNA-seq of cartilage reveals an over-abundance of miR-140-3p which contains functional isomiRs — microRNA-seq of cartilage reveals an overabundance of miR-140-3p which contains functional isomiRs — Supplemental Material 

# microRNA-seq of cartilage reveals an overabundance of miR-140-3p which contains functional isomiRs

## Supplemental Material

- Supplemental\_Figures.pdf
- Supplemental\_Legends.docx
- Supplemental\_Tables.xlsx
