# Supplementary material for: microRNA-seq of cartilage reveals an overabundance of miR-140-3p which contains functional isomiRs
Source: RNA. 2020 Nov;26(11):1575–88. doi: 10.1261/rna.075176.120 (PMC7566571; doi:10.1261/rna.075176.120)
Supplement: Supplemental Material [file supp_075176.120_Supplemental_Figures.pdf]

Figure S1

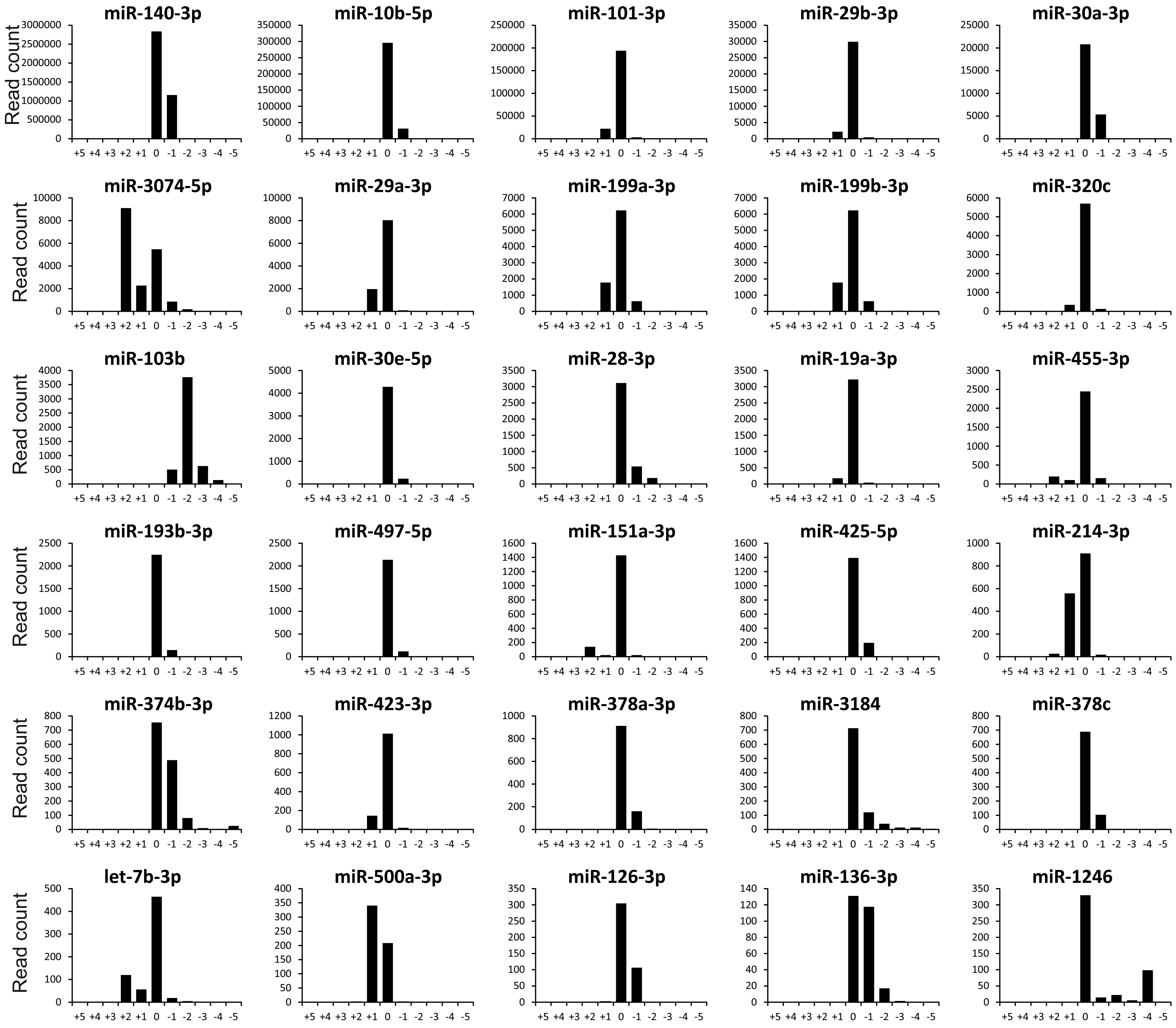
miR-140-3p
miR-10b-5p
miR-101-3p
miR-29b-3p
miR-30a-3p
miR-3074-5p
miR-29a-3p
miR-199a-3p
miR-199b-3p
miR-320c
miR-103b
miR-30e-5p
miR-28-3p
miR-19a-3p
miR-455-3p
miR-193b-3p
miR-497-5p
miR-151a-3p
miR-425-5p
miR-214-3p
miR-374b-3p
miR-423-3p
miR-378a-3p
miR-3184
miR-378c
let-7b-3p
miR-500a-3p
miR-126-3p
miR-136-3p
miR-1246
Read count
+5 +4 +3 +2 +1 0 -1 -2 -3 -4 -5

Figure S2

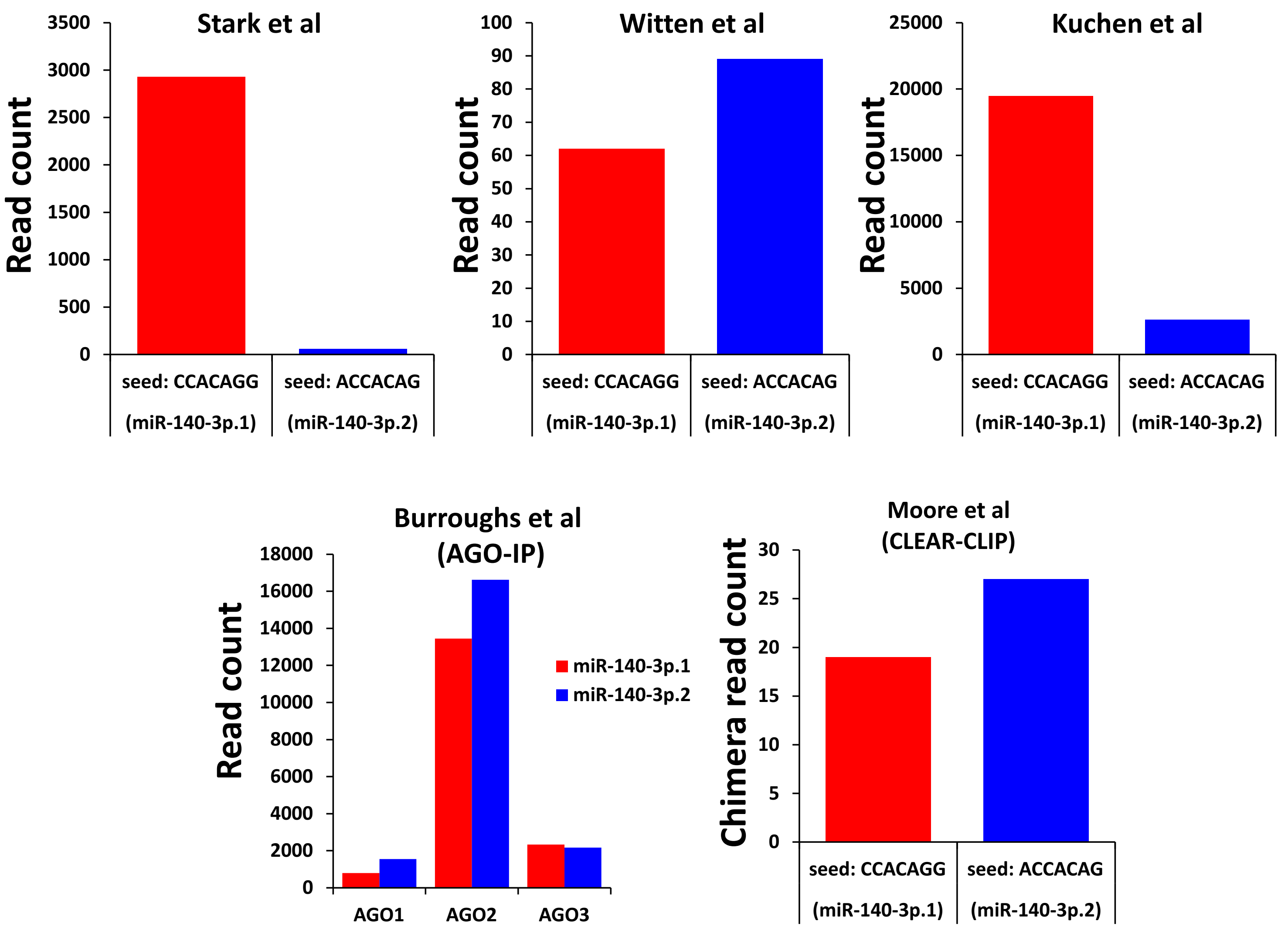
Stark et al
Read count
3500
3000
2500
2000
1500
1000
500
0
seed: CCACAGG
(miR-140-3p.1)
seed: ACCACAG
(miR-140-3p.2)
Witten et al
Read count
100
90
80
70
60
50
40
30
20
10
0
seed: CCACAGG
(miR-140-3p.1)
seed: ACCACAG
(miR-140-3p.2)
Kuchen et al
Read count
25000
20000
15000
10000
5000
0
seed: CCACAGG
(miR-140-3p.1)
seed: ACCACAG
(miR-140-3p.2)
Burroughs et al
(AGO-IP)
Read count
18000
16000
14000
12000
10000
8000
6000
4000
2000
0
miR-140-3p.1
miR-140-3p.2
AGO1
AGO2
AGO3
Moore et al
(CLEAR-CLIP)
Chimera read count
30
25
20
15
10
5
0
seed: CCACAGG
(miR-140-3p.1)
seed: ACCACAG
(miR-140-3p.2)

Figure S3

	Source	Term Name	Term ID	Term size	Intersection	Adjusted P-value
**A**	**GO:MF**	**extracellular matrix structural constituent conferring tensile strength**	**GO:0030020**	**41**	**5**	**0.04464093**
B	GO:BP	anatomical structure morphogenesis	GO:0009653	2820	63	0.001590813
C	GO:BP	decidualization	GO:0046697	27	6	0.001696492
**D**	**GO:BP**	**connective tissue development**	**GO:0061448**	**281**	**15**	**0.004617556**
E	GO:BP	maternal placenta development	GO:0001893	38	6	0.01414184
F	GO:BP	double-strand break repair via break-induced replication	GO:0000727	11	4	0.015265416
G	GO:BP	tube development	GO:0035295	1143	32	0.021966518
H	REAC	Unwinding of DNA	REAC:R-HSA-176974	12	4	0.005710503
**I**	**REAC**	**Assembly of collagen fibrils and other multimeric structures**	**REAC:R-HSA-2022090**	**60**	**7**	**0.006117096**

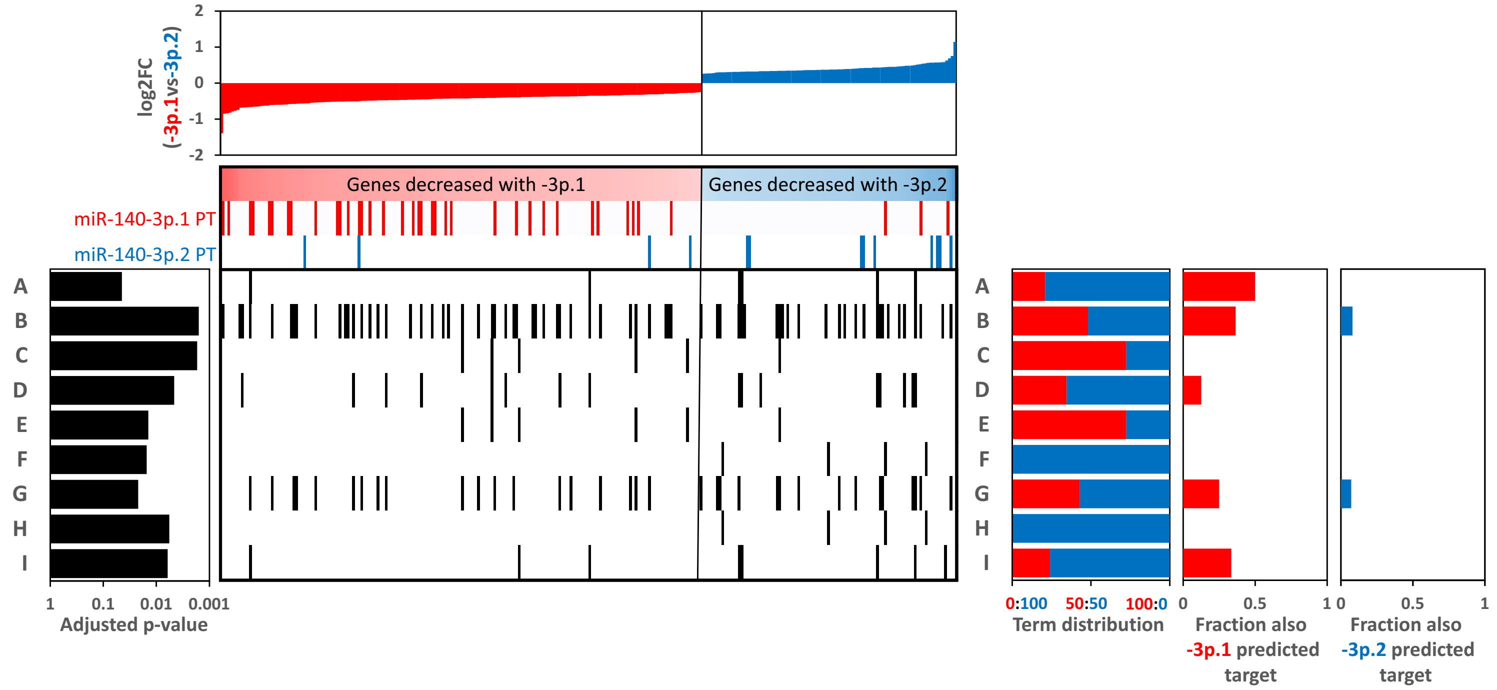

Figure S4

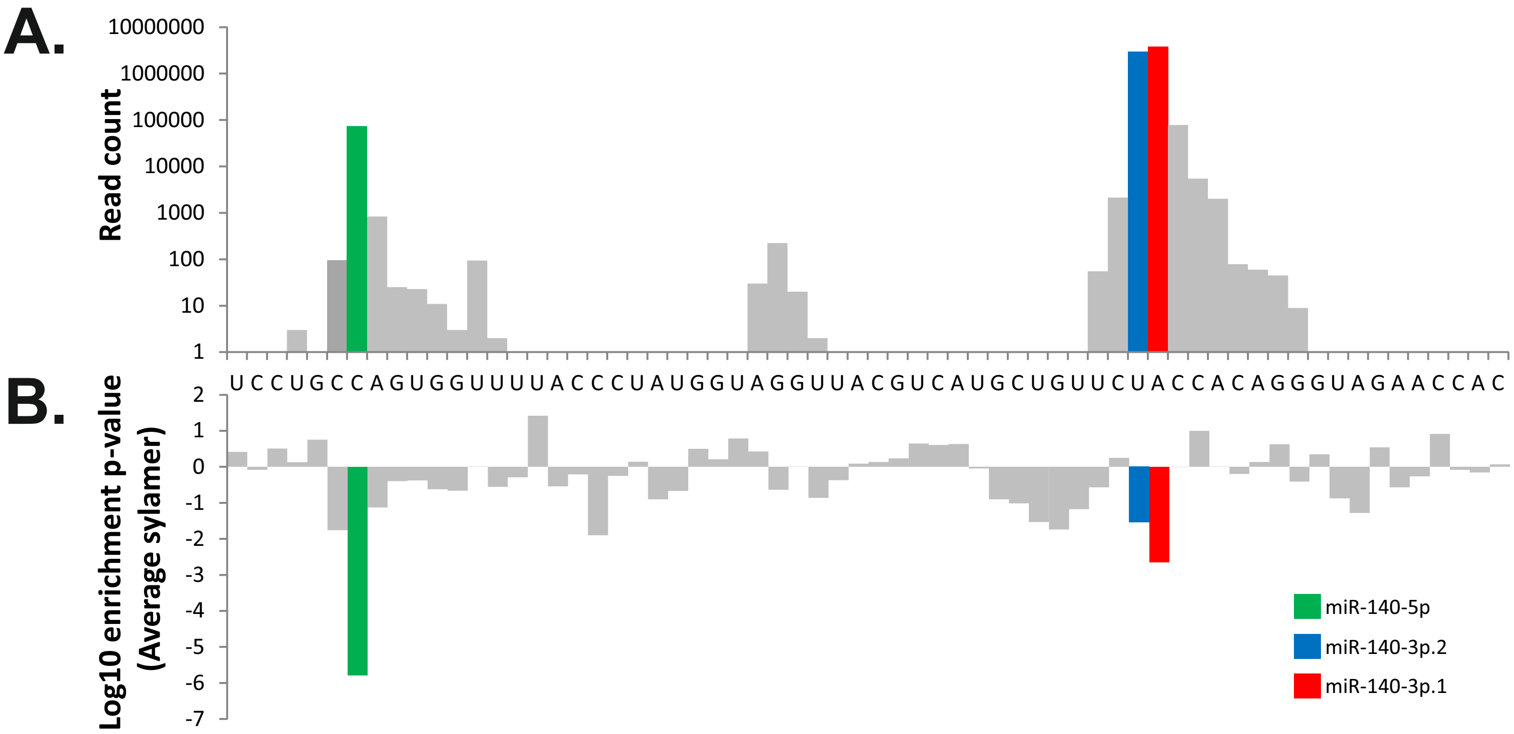
A.
B.
Read count
10000000
1000000
100000
10000
1000
100
10
1
U C C U G C C A G U G G U U U U A C C C U A U G G U A G G U U A C G U C A U G C U G U U C U A C C A C A G G G U A G A A C C A C
Log10 enrichment p-value
(Average sylamer)
2
1
0
-1
-2
-3
-4
-5
-6
-7
miR-140-5p
miR-140-3p.2
miR-140-3p.1

Figure S5

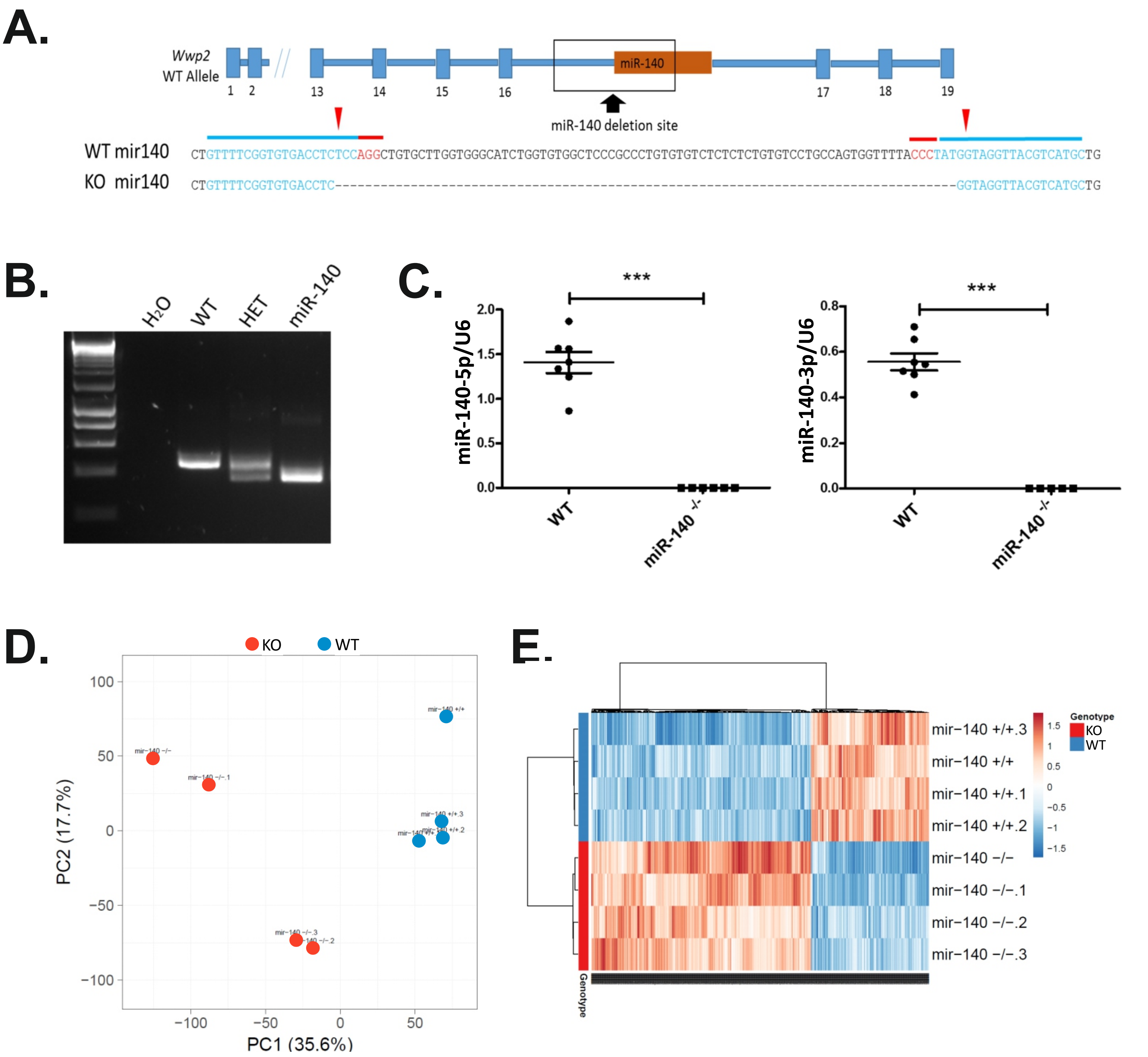
A.
Wwp2
WT Allele
1
2
13
14
15
16
miR-140
17
18
19
miR-140 deletion site
WT mir140
CTGTTTTCGGTGTGACCTCTCCAGGCTGTGCTTGGTGGGCATCTGGTGTGGCTCCCGCCCTGTGTGTCTCTCTCTGTGTCCTGCCAGTGGTTTTACCCTATGGTAGGTTACGTCATGCTG
KO mir140
CTGTTTTCGGTGTGACCTC-----GGTAGGTTACGTCATGCTG
B.
H2O
WT
HET
miR-140
C.
***
miR-140-5p/U6
2.0
1.5
1.0
0.5
0.0
WT
miR-140 -/-
***
miR-140-3p/U6
0.8
0.6
0.4
0.2
0.0
WT
miR-140 -/-
D.
KO
WT
100
50
0
−50
−100
PC2 (17.7%)
−100
−50
0
50
PC1 (35.6%)
E.
mir−140 +/+.3
mir−140 +/+
mir−140 +/+.1
mir−140 +/+.2
mir−140 −/−
mir−140 −/−.1
mir−140 −/−.2
mir−140 −/−.3
Genotype
1.5
1
0.5
0
−0.5
−1
−1.5
Genotype
KO
WT

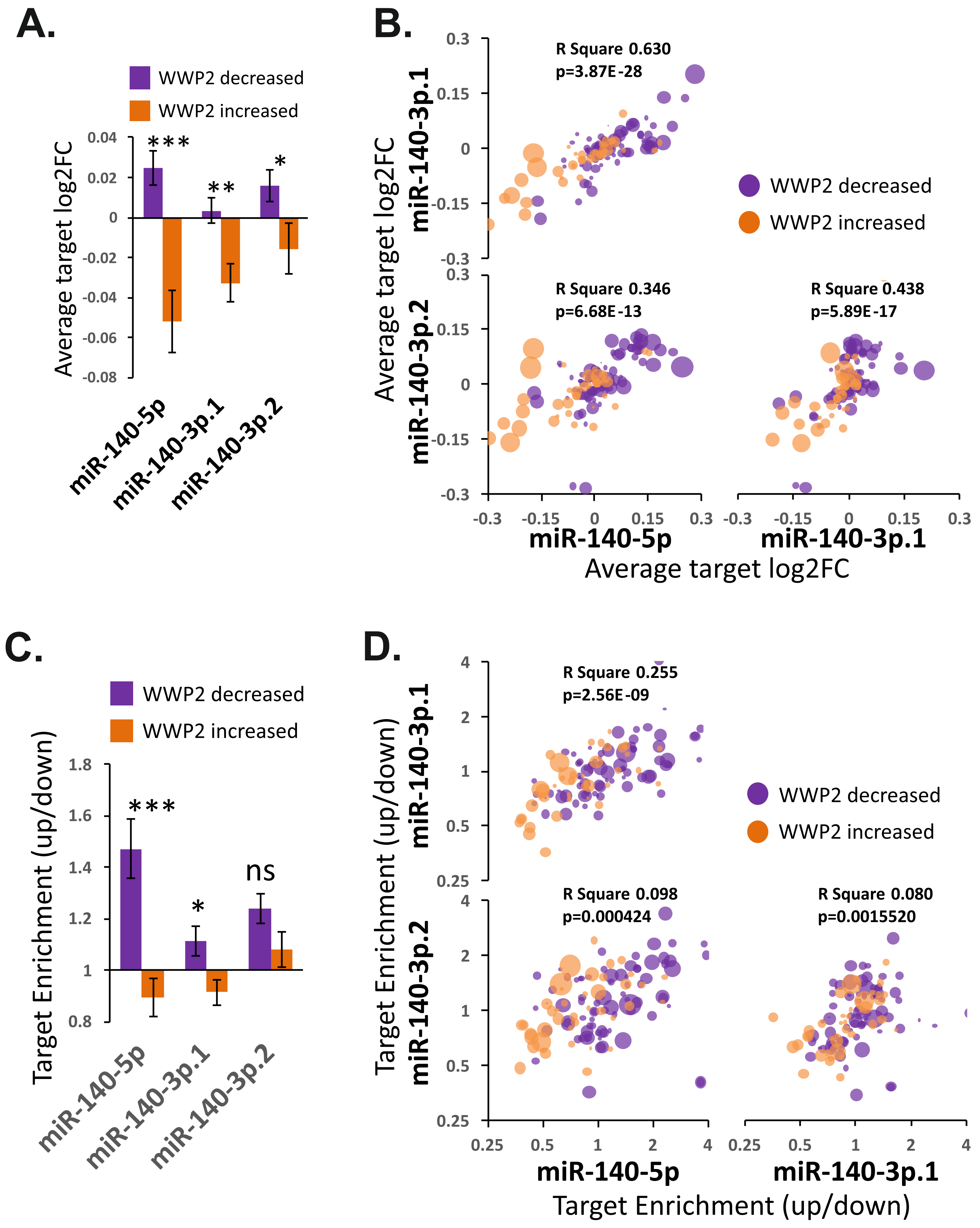
A.
WWP2 decreased
WWP2 increased
Average target log2FC
0.04
0.02
0
-0.02
-0.04
-0.06
-0.08
***
**
*
miR-140-5p
miR-140-3p.1
miR-140-3p.2
B.
R Square 0.630
p=3.87E-28
R Square 0.346
p=6.68E-13
R Square 0.438
p=5.89E-17
0.3
0.15
0
-0.15
-0.3
Average target log2FC
miR-140-3p.1
miR-140-3p.2
WWP2 decreased
WWP2 increased
-0.3
-0.15
0
0.15
0.3
miR-140-5p
miR-140-3p.1
Average target log2FC
C.
WWP2 decreased
WWP2 increased
Target Enrichment (up/down)
1.8
1.6
1.4
1.2
1
0.8
***
*
ns
miR-140-5p
miR-140-3p.1
miR-140-3p.2
D.
R Square 0.255
p=2.56E-09
R Square 0.098
p=0.000424
R Square 0.080
p=0.0015520
4
2
1
0.5
0.25
Target Enrichment (up/down)
miR-140-3p.1
miR-140-3p.2
WWP2 decreased
WWP2 increased
0.25
0.5
1
2
4
miR-140-5p
miR-140-3p.1
Target Enrichment (up/down)

Figure S7

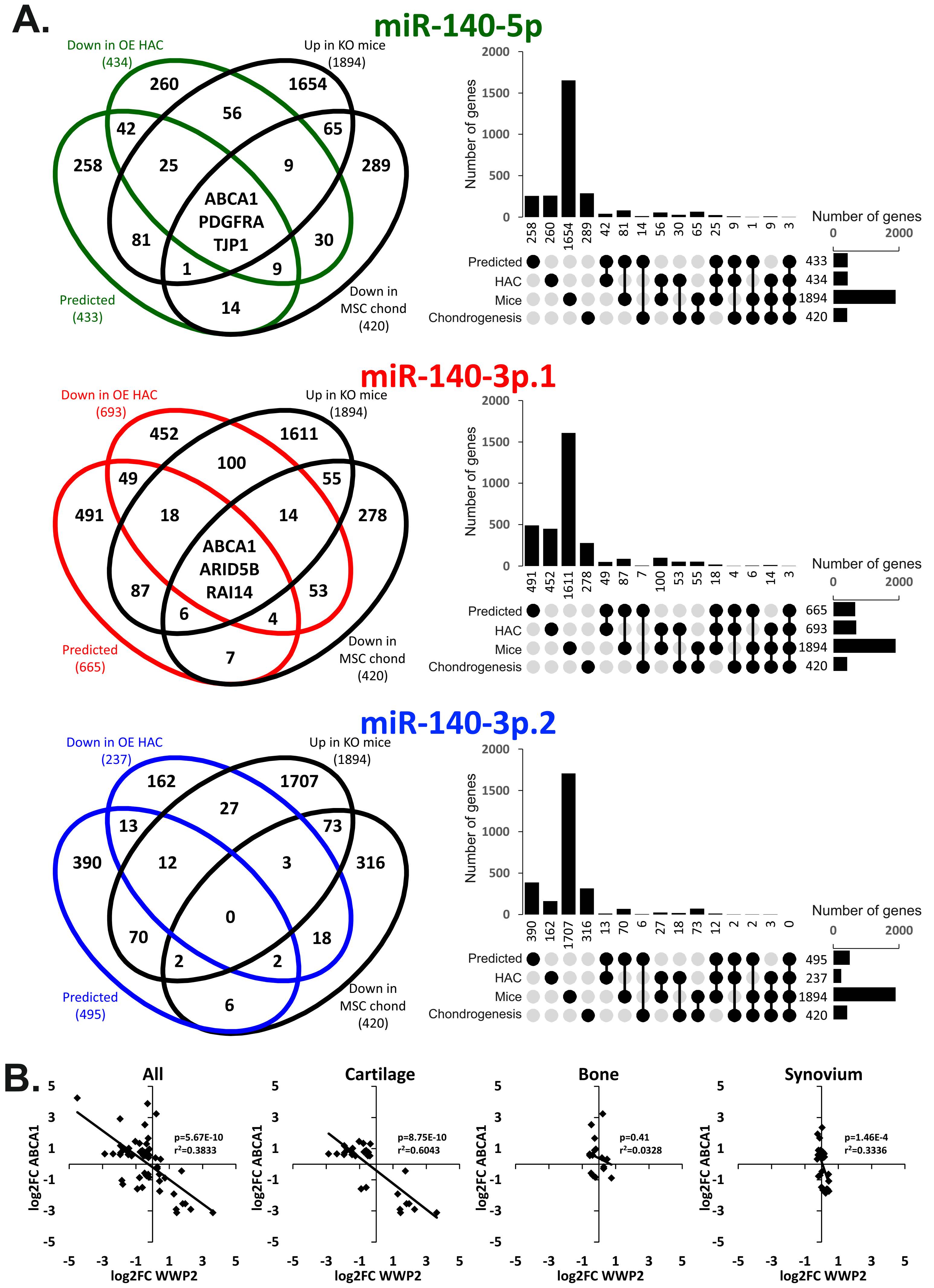
A.
miR-140-5p
Down in OE HAC (434)
Up in KO mice (1894)
Predicted (433)
Down in MSC chond (420)
260
1654
56
42
65
258
25
9
289
ABCA1
PDGFRA
TJP1
81
30
1
9
14
Number of genes
2000
1500
1000
500
0
258 260 1654 289 42 81 14 56 30 65 25 9 1 9 3
Predicted 433
HAC 434
Mice 1894
Chondrogenesis 420
miR-140-3p.1
Down in OE HAC (693)
Up in KO mice (1894)
Predicted (665)
Down in MSC chond (420)
452
1611
100
49
55
491
18
14
278
ABCA1
ARID5B
RAI14
87
53
6
4
7
491 452 1611 278 49 87 7 100 53 55 18 4 6 14 3
Predicted 665
HAC 693
Mice 1894
Chondrogenesis 420
miR-140-3p.2
Down in OE HAC (237)
Up in KO mice (1894)
Predicted (495)
Down in MSC chond (420)
162
1707
27
13
73
390
12
3
316
0
70
18
2
2
6
390 162 1707 316 13 70 6 27 18 73 12 2 2 3 0
Predicted 495
HAC 237
Mice 1894
Chondrogenesis 420
B.
All
p=5.67E-10
r²=0.3833
Cartilage
p=8.75E-10
r²=0.6043
Bone
p=0.41
r²=0.0328
Synovium
p=1.46E-4
r²=0.3336
log2FC ABCA1
log2FC WWP2
-5 -3 -1 1 3 5
