# Supplementary material for: microRNA-seq of cartilage reveals an overabundance of miR-140-3p which contains functional isomiRs
Source: RNA. 2020 Nov;26(11):1575–88. doi: 10.1261/rna.075176.120 (PMC7566571; doi:10.1261/rna.075176.120)
Supplement: Supplemental Material [file supp_075176.120_Supplemental_Legends.docx]

**SUPPLEMENTAL FIGURE LEGENDS**

**Supplemental Figure S1.** Cartilage miRNAs which have abundant 5´ isomiRs. Twenty-nine miRNAs with isomiRs that account for more than 5% of the reads for that miRNA and the number of isomiR reads is greater than 100. miR-1246 isomiR (read count 99) is also shown. 5´ start position of each isomiR relative to the miRBase annotation is shown: the addition of a nucleotide is designated as ‘+1’ and the loss as ‘-1’, ‘0’ represents reads of the miRBase annotation. Histogram indicates read count.

**Supplemental Figure S2.** Analysis of published RNA-seq. miR-140-3p.1 and miR-140-3p.2 were present in multiple sRNA-seq data including; melanoma ([Stark et al. 2010](#_ENREF_51)), cervix ([Witten et al. 2010](#_ENREF_55)), lymphocytes ([Kuchen et al. 2010](#_ENREF_28)), and following immunoprecipitation of Argonaute (AGO) proteins ([Burroughs et al. 2011](#_ENREF_10)). Sequences were designated either miR-140-3p.1 or miR-140-3p.2 based upon seed sequence. The isomiRs were also approximately equally detected in CLEAR-CLIP data from mouse cortex tissue ([Moore et al. 2015](#_ENREF_38)), however their low expression in this tissue did not allow for target prediction from the covalently ligated mRNAs.

**Supplemental Figure S3.** Pathway analysis of differentially expressed genes using g-Profiler. Pathway analysis of the 237 genes differentially expressed between miR-140-3p.1 and miR-140-3p.2 transfected chondrocytes using g-Profiler. Significant gene ontology molecular function (G0:MF), biological processes (GO:BP) and Reactome (REAC) terms are shown. The adjusted p-values, distribution within terms between downregulation with each isomiR and the fraction of these genes that are also predicted targets of miR-140-3p.1 and miR-140-3p.2 are depicted. Distribution of miR-140-3p.1 and miR-140-3p.2 TargetScan predicted targets (PT) are also shown.

**Supplemental Figure S4.** IsomiR read count and enrichment score for all possible sequences derived from miR-140. (A) Read count for all 5´isomiRs encoded from the mouse miR-140 stem loop according to miRBase. (B) Sylamer analysis of rib cartilage RNA from mice lacking miR-140 stem loop for 7m8 seed binding sites corresponding to every possible miRNA produced from the *Mir140* locus. Histogram represents average enrichment score for each seed binding motif.

**Supplemental Figure S5.** Validation of miR-140 null mouse model and rib chondrocyte RNA-seq. (A) Deletion of *Mir140* was attained using the CRISPR/-Cas9 system. The *Mir140* locus was targeted in mouse zygotes using two crRNAs (blue line, protospacer adjacent motif (PAM) shown in red). This successfully deleted the *Mir140* locus sequence depicted (KO mir140). (B) Mice were bred to homozygosity with the genotype in wild-type, heterozygous and null mice confirmed through PCR of ear-notch genomic RNA. (C) Total RNA was extracted from rib chondrocytes of postnatal day 7 (P7) mice, reverse transcribed to cDNA and subjected to real time qRT-PCR analysis for gene expression of miR-140-5p and miR-140-3p (canonical). Values were normalised to U6 and plotted as mean ± standard error of the mean (SEM) (number of samples, WT=7, miR-140^-/-^ =6). *** p≤0.001. (D) The RNA from several of the same samples as (C) was subjected to RNA-seq (n=4 for WT and miR-140^-/-^). Principle component analysis of length-scaled TPM (Transcripts Per Million) values segregated WT and KO (blue and red). (E) Heat map of the 1200 most differentially expressed genes.

**Supplemental Figure S6.** Comparison of miR-140-5p, miR-140-3p.1 and miR-140-3p.2 target fold change and enrichment in studies where *WWP2* expression either decrease or increases. (A) Average log2FC for miR-140-5p, miR-140-3p.1 and miR-140-3p.2 targets within studies where *WWP2* expression either decreased for increased. (B) Correlation between average log2FC for miR-140-5p, miR-140-3p.1 and miR-140-3p.2 predicted targets for all studies where *WWP2* expression changed. (C) Enrichment for miR-140-5p, miR-140-3p.1 and miR-140-3p.2 targets within studies where *WWP2* either decreased or increased. (D) Correlation between enrichment for miR-140-5p, miR-140-3p.1 and miR-140-3p.2 predicted targets for all studies where *WWP2* expression changed. Studies where *WWP2* significantly decreased are shown in purple and increased shown in orange. For (B) and (C) symbol size if proportional to the level of *WWP2* change in expression. The enrichment was calculated by dividing the percentage of targets within upregulated genes by the percentage of targets within downregulated genes for each study.

**Supplemental Figure S7.** Overlap between human and mouse miR140 datasets. (A) overlap between predicted targets, genes downregulated following overexpression in human articular chondrocytes (HAC), genes upregulated in mice lacking the *Mir140* locus and genes downregulated during human MSC chondrogenesis. Data represented as Venn diagram and ‘UpSet’ plot. (B) Regression analysis of *ABCA1* expression and *WWP2* in multiple skeletal tissues, separated based on tissue type.

**SUPPLEMENTAL TABLE LEGENDS**

**Supplemental Table S1. Cartilage sRNA-seq.** Cartilage sRNA-seq from 3 donors (c15, c16 and c17). All sequences including isomiRs aligning to 990 mature miRNAs are shown with read count.

**Supplemental Table S2. Predicted targets.** Predicted targets of miR-140-5p, miR-140-3p.1 and miR-140-3p.2 by TargetScanHuman 7.2. Gene lists correspond to Venn diagram in figure 2B.

**Supplemental Table S3. Human chondrocyte gene expression.** Human chondrocyte gene expression changes following transfection of miRNA mimics: miR-140-3p.1 vs CON, miR-140-3p.2 vs CON, miR-140-3p.1 vs miR-140-3p.2

**Supplemental Table S4. Human chondrocyte gene list.** Gene lists from human chondrocytes: Genes decreased (down) with miR-140-3p.1 vs control, Genes decreased (down) with miR-140-3p.2 vs control, Genes decreased (down) with both miR-140-3p.1 and miR-140-3p.2 vs control, and genes significantly different between miR-140-3p.1 and miR-140-3p.2 when directly compared.

**Supplemental Table S5. Human chondrocyte pathway analysis.** Pathway analysis (PA) using DAVID for genes decreased with miR-140-3p.1, genes decreased with miR-140-3p.2 genes significantly different between miR-140-3p.1 and miR-140-3p.2.

**Supplemental Table S6. *Mir140* KO mice gene expression**. RNA-seq of mouse rib cartilage of *Mir140* KO mice and WT control mice

**Supplemental Table S7. *Mir140* KO mice gene lists.** List of up and downregulated genes in *Mir140* KO mice

**Supplemental Table S8. *Mir140* KO mice pathway analysis**. Pathway analysis using DAVID for genes upregulated in *Mir140* KO mice

**Supplemental Table S9. Human vs mouse.** Cross over between predicted targets, human chondrocyte data and mouse *Mir140* KO data

**Supplemental Table S10. Oligonucleotides.** Table of oligonucleotides used in this study. Targets of miR-140-3p.1 and miR-140-3p.2 used for cloning into pMiR-GLO by InFusion. Primers used for the generation and genotyping of miR-140^-/-^ mice.
